# Influence of *Bacillus subtilis*-Instigated Calcite Precipitation on Damage Progression and Ionic Transport

**DOI:** 10.3390/ma19061153

**Published:** 2026-03-16

**Authors:** Sana Gul, Nafeesa Shaheen

**Affiliations:** 1 Laboratory for Structural Engineering, Institute of Material Testing and Building Materials Technology, Graz University of Technology, 8010 Graz, Austria; 2 Department of Civil Engineering, National University of Technology, I-12, Islamabad 44000, Pakistan

**Keywords:** post-cracking damage evolution, transport resistance, flexural toughness, chloride ion penetration, capillary sorptivity, microbial-induced calcium carbonate precipitation (MICP)

## Abstract

Bacteria-based self-healing concrete is extensively shown to improve strength and durability; yet, the mechanistic relationship among microbial activity, damage progression, and transport resistance is still ambiguous. This study examines the interrelated mechanical and transport properties of concrete that incorporates *Bacillus subtilis* by directly substituting mixing water. Concrete mixtures with 0%, 5%, and 10% bacterial solution were assessed for compressive strength, complete stress–strain response, split tensile strength, flexural toughness, fast chloride ion penetration, and capillary sorptivity. X-ray diffraction was employed for microstructural validation. Results indicate a dose-dependent shift from brittle to quasi-ductile behavior, marked by augmented strain capacity, postponed crack localization, and improved post-cracking energy absorption. The mechanical alterations resulted in substantial decreases in chloride ion penetrability (up to 57%) and capillary sorptivity (up to 60%), signifying a drop in crack-assisted transport. X-ray diffraction verified the production of calcite resulting from microbial-induced calcium carbonate precipitation. The results indicate that the improvement in durability of bacterial concrete is attributable not only to pore filling but also to altered damage mechanisms that diminish the connectedness of transport channels, underscoring the potential of *Bacillus subtilis* as a bio-admixture for resilient structural concrete.

## 1. Introduction

Cracking in concrete is an unavoidable occurrence caused by plastic shrinkage, thermal stresses, drying shrinkage, and mechanical pressure [1]. These cracks allow aggressive chemicals like chloride ions, sulfates, and moisture to accelerate reinforcement degradation and reduce durability [1,2]. Conventional repair techniques, including epoxy injection and surface sealing, are often expensive, labor-intensive, and environmentally unfriendly, motivating the development of autonomous self-healing technologies for cementitious materials [3,4].

Self-healing concrete technologies can be broadly classified into autogenous, chemical, and biological approaches [5]. Autogenous healing relies on continued hydration of unreacted cement particles and carbonation of calcium hydroxide [6]; however, its effectiveness is limited to small crack widths and favorable environmental conditions [3]. Chemical encapsulation techniques, involving the incorporation of healing agents within capsules or vascular networks [7], have demonstrated improved crack sealing capability but are often constrained by high cost, limited compatibility with the cement matrix, and reduced long-term efficiency [7,8].

Biological self-healing using microorganisms has emerged as a promising and sustainable alternative. Among various biological mechanisms, microbial-induced calcium carbonate precipitation (MICP) has attracted significant attention due to its ability to autonomously seal cracks and improve durability [9]. In MICP, bacteria metabolize nutrient sources to produce carbonate ions, which react with calcium ions present in the cement matrix to form calcium carbonate that fills pores and cracks [10,11,12]. This process not only seals cracks but also densifies the microstructure, thereby enhancing mechanical strength and reducing permeability. Furthermore, because of its high urease activity, *Sporosarcina pasteurii*, a well-known ureolytic bacterium that has been thoroughly investigated for microbial-induced calcium carbonate precipitation, has shown notable mechanical strength recovery and quick crack sealing (up to about 0.4 mm) [13]. In cementitious materials, *Bacillus megaterium* has also been shown to increase compressive and flexural strengths, improve crack-healing effectiveness, and decrease permeability [14]. Other calcite-precipitating *Bacillus* species have also shown notable reductions in water absorption and chloride ion penetration, further highlighting the broader applicability of different bacterial strains for durability enhancement in concrete structures [13,14,15,16]. These findings indicate that different microbial species can successfully contribute to strength increase and crack repair, with overall performance governed by bacterial survivability in alkaline environments and calcium carbonate precipitation efficiency.

Spore-forming bacteria from the *Bacillus* genus, particularly *Bacillus subtilis*, are widely regarded as suitable candidates for bio-concrete applications due to their ability to survive harsh alkaline environments and remain dormant for extended periods [17,18]. Upon crack formation and moisture ingress, bacterial spores become metabolically active, initiating calcite precipitation within the crack volume [19]. Several studies have reported notable improvements in compressive, tensile, and flexural strengths of concrete incorporating *Bacillus subtilis* [20,21]. Strength enhancements ranging from 15% to 50% have been attributed to pore refinement and improved interfacial transition zones resulting from calcite deposition [22,23,24].

Beyond strength enhancement, bacterial concrete has demonstrated improved durability performance. Refs. [25,26] reported significant reductions in chloride ion penetration and water absorption in *Bacillus*-based concretes, highlighting their potential for corrosion-resistant infrastructure. Similarly, reductions in capillary sorptivity of up to 60% have been observed due to pore blocking by microbial calcite [27,28]. These durability improvements are particularly relevant for concrete structures exposed to marine environments or de-icing salts.

Despite these promising findings, existing studies exhibit several limitations. Many investigations focus primarily on compressive strength or crack-healing efficiency, with limited attention to cracking behavior, energy absorption, and toughness properties critical for structural applications and resistance to dynamic or seismic loading [29]. Furthermore, a significant portion of the literature employs encapsulation or immobilization techniques for bacterial incorporation, which may hinder large-scale implementation due to increased material cost and processing complexity [30]. Another underexplored aspect is the influence of bacterial dosage introduced through direct replacement of mixing water on both mechanical and transport properties. While direct mixing methods offer simplicity and cost-effectiveness, systematic studies linking bacterial concentration to strength, toughness, and durability remain scarce [31,32]. Moreover, limited research has combined mechanical testing with transport property evaluation and microstructural validation to establish a comprehensive understanding of bacterial concrete performance.

In light of these gaps, the present study aims to advance the current understanding of *Bacillus subtilis*-based self-healing concrete by employing a direct water-replacement strategy and conducting an integrated evaluation of mechanical performance, post-cracking behavior, durability, and microstructural characteristics. This approach seeks to provide practical insights into the feasibility of bacterial concrete for sustainable and durable structural applications.

### Novelty and Contribution of the Present Study

This study describes how microbial-induced calcium carbonate precipitation (MICP), post-peak mechanical behavior, and transport features in bacteria-based self-healing concrete interact rather than reporting isolated strength or durability increases. Unlike most previous research on crack closure efficiency or compressive strength recovery, *Bacillus subtilis* dramatically influences damage evolution and pore-connectivity pathways, driving mechanical deterioration and chlorine transport in concrete. Using total stress–strain response, fracture propagation patterns, and flexural toughness indices, this study found a dose-dependent transition from brittle to quasi-ductile compressive behavior. Bacteria enhance matrix density, delay crack localization, promote distributed microcracking, and dissipate post-peak energy, limiting transport channels. By assessing compressive and flexural toughness, chloride ion penetrability, and capillary sorptivity, this study relates post-cracking mechanical response and transport resistance in bacterial concrete. Microstructural reconfiguration reduces crack-assisted permeability and increases durability, a *Bacillus*-based concrete process not previously found. Direct water replacement is realistic and allows mechanistic interpretation without encapsulation. Calcite generation in X-ray diffraction supports MICP-driven pore refinement and interfacial transition zone densification, which affect strength–ductility–durability synergy. This integrated mechanical–transport framework highlights *Bacillus subtilis*’ value as a bio-admixture for durable structural concrete.

## 2. Materials and Methods

### 2.1. Constituent Concrete Materials

This study prepared *Bacillus subtilis* concrete using ordinary Portland cement (OPC) CEM-1 of 53 grade as the major binding agent due to its weather resistance and aggregate binding. The chemical and physical composition of OPC is given in Table 1. The chemical composition in oxide form was determined using X-ray fluorescence (XRF) spectroscopy (S8 TIGER, Bruker, Karlsruhe, Germany).

Properties of aggregates are tabulated in Table 2.

### 2.2. Germination of Bacteria

**Figure 1:**
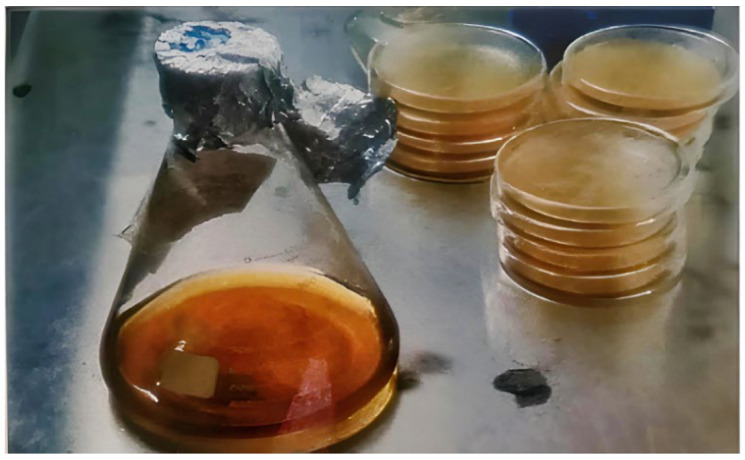
Preparation of the bacterial culture in the liquid medium (Luria–Bertani broth) at 37 °C for 24 h.

Table 3 presents the composition of the *Bacillus subtilis* combination. A spore-forming bacterium was chosen for its potential as a self-repairing agent in concrete. *Bacillus subtilis* is recognized for its robustness under extreme alkaline conditions and its capacity to facilitate calcium carbonate precipitation [18,35]. Microbial culturing was carried out as per the standard procedure [10]. The microorganism was cultured in a nutrient-dense medium formulated with Luria–Bertani (LB) broth. The medium’s composition comprised tryptone (2.5 g/L) as a nitrogen source for amino acids and peptides vital for protein synthesis and bacterial proliferation, sodium chloride (2.5 g/L) to preserve osmotic equilibrium and facilitate cellular functions, and yeast extract (1.75 g/L) to furnish essential vitamins, minerals, and growth factors that augment bacterial metabolism. These components were solubilized in 25 g of distilled water, with the addition of 1 g of LB broth powder to establish a conducive environment for optimal bacterial proliferation. The liquid medium was incubated statically at 37 °C for 24 h to attain the desired bacterial concentration, as shown in Figure 1. The bacterial cell content in the medium was quantified by optical density measurements at 600 nm, and the final suspension was calibrated to attain a concentration of 1.9 × 10^7^ cells/cm^3^ of concrete. This concentration was chosen based on previously reported effective bacterial densities for microbial-induced calcium carbonate precipitation (MICP) in cementitious materials, which typically range between 10^6^ and 10^8^ cells/mL, ensuring sufficient biomineralization activity while maintaining bacterial survivability in the alkaline concrete environment [32,36,37]. The self-healing additive was formulated using *Bacillus subtilis* and calcium lactate, which acted as a nutrition precursor to maintain bacterial viability and facilitate successful calcium carbonate precipitation inside the concrete matrix. The liquid medium was incubated statically at 37 °C for 24 h to attain the desired bacterial concentration.

Because *Bacillus subtilis* is a non-ureolytic bacterium that produces calcium carbonate precipitation through metabolic activity as compared to urea hydrolysis, calcium lactate was chosen as the nutritional supply. In this metabolic method, calcium lactate functions as a readily available organic carbon source that bacteria can metabolically transform into carbonate ions, which then react with calcium ions to precipitate CaCO_3_ within the cement matrix. The utilization of calcium lactate is beneficial as it prevents the production of ammonia linked to ureolytic systems that rely on urea hydrolysis, which can negatively impact concrete durability and reinforcing integrity [38,39].

**Figure 2:**
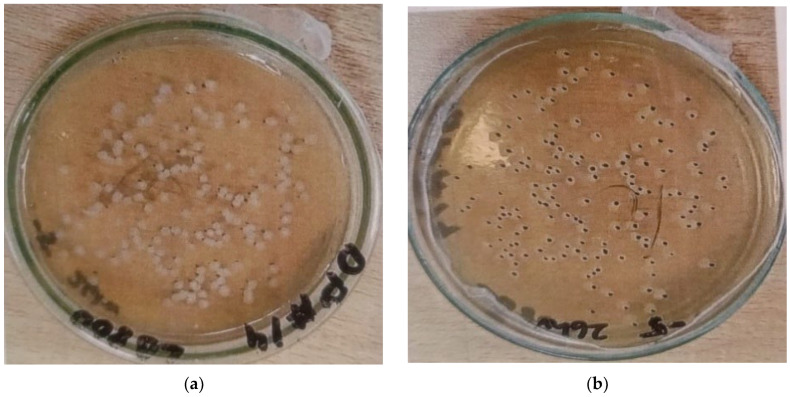
(**a**) Growth of *Bacillus subtilis* colonies on nutrient-rich agar medium prepared with tryptone, NaCl, yeast extract, and LB broth, showing characteristic white, (**b**) round colonies indicative of successful bacterial cultivation.

Optical density was quantified with a spectrophotometer to evaluate the turbidity of the bacterial culture at a wavelength of 600 nanometers (nm) [40]. Turbidity exhibited a direct correlation with bacterial content in the medium, with elevated optical density (OD) values signifying increased bacterial density, as demonstrated in Figure 2. After determining the bacterial concentration, the culture was modified to attain the desired concentration of 1.9 × 10^7^ bacterial cells per cubic centimeter (cells/cm^3^). This guaranteed the appropriate quantity of bacteria was included in the concrete mixture to facilitate effective self-healing.

### 2.3. Mixing Regime

Table 4 shows the concrete mix design. There were 54 cylindrical specimens (150 mm height, 100 mm diameter) and 27 beamlets (100 mm × 100 mm × 500 mm) used to evaluate compressive, split tensile, and flexural strength. A laboratory mixer was used to mix concrete according to ASTM C192 [41]. First, coarse and fine particles were dry mixed for 1 min to disperse evenly. Ordinary Portland cement was added, and dry mixing continued for another minute. While mixing for 2 min, the mixing water or bacterial solution was gradually added. The *Bacillus subtilis* solution replaced 5% and 10% of the total mixing water by weight in bacterial concrete compositions, while the control mixture used tap water. Calcium lactate was incorporated as a nutrient source at 1% of the cement weight, consistent with the dosage used in previous studies [32], which demonstrated that this concentration provides adequate nutrients for bacterial metabolism and enhances calcium carbonate precipitation responsible for crack healing in concrete. For a consistent and workable consistency, the mixture was stirred for 2 min after adding the liquid. No formulation was mixed longer than 6 min. All combinations had a 0.50 water-to-cement ratio to isolate bacterial integration. As used in bacterial concrete research, direct mixing was chosen for its simplicity and usefulness in large-scale concrete manufacture [31]. All combinations were consistent visually, with no segregation or bleeding during casting.

## 3. Fresh Properties

### 3.1. pH of Fresh Concrete Containing Bacillus subtilis

**Figure 3:**
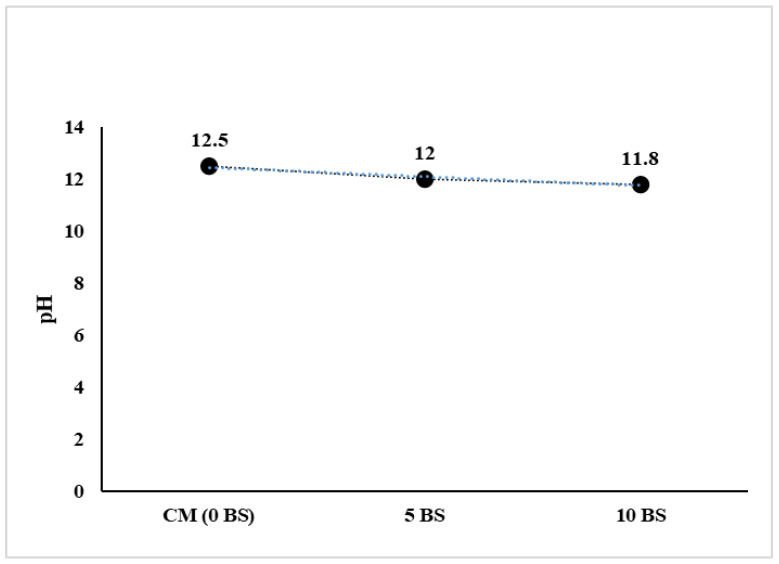
pH of concrete with and without *Bacillus subtilis*.

Microbial growth occurs within a certain pH tolerance range, with optimal metabolic activity generally observed near neutral circumstances (pH 6.5–7.5) and survival feasible across a wider range of approximately pH 4–9. The specified pH limits indicate the physiological adaptation of bacteria to their natural habitats and the stability of cellular enzymes and membranes [31]. Conversely, freshly mixed concrete presents a significantly alkaline environment (pH = 12–13), chiefly because of the dissolution of calcium hydroxide produced during cement hydration. The pH values of the fresh concrete mixtures in this investigation were 12.5 for the control mix and somewhat lower at 12.0 and 11.8 for the 5% and 10% *Bacillus subtilis* mixes, respectively, remaining significantly over the ideal pH range for active bacterial growth, as shown in Figure 3. This signifies that *Bacillus subtilis* put into the fresh concrete does not promptly engage in aggressive metabolic growth. The bacteria are anticipated to remain primarily in a dormant or spore state, aligning with the established alkaliphilic tolerance and spore-forming capacity of *Bacillus* species. The minor decrease in pH associated with elevated bacterial dosage does not suggest conditions conducive to active bacterial proliferation; instead, it signifies chemical interactions between the bacterial solution and alkaline cementitious components, such as partial buffering or hydroxyl ion consumption. Previous studies [42] have reported analogous findings, indicating that concrete retains a high alkalinity (pH ≈ 11–13) that inhibits bacterial growth unless intentional buffering is implemented, while still preserving the necessary alkalinity for durability and reinforcement passivation. While the fresh concrete environment is inhospitable for immediate bacterial proliferation, it is conducive to long-term bacterial survival in a latent state. Crack formation and moisture infiltration over the service life of concrete can lead to localized pH drop and increased water availability, which activate bacterial metabolism, facilitating microbial-induced calcium carbonate precipitation and self-healing [11]. This relationship elucidates how *Bacillus subtilis* can be integrated into concrete without detriment to fresh-state performance while maintaining viability for prolonged healing efficacy.

To better understand the long-term survival strategy, it is crucial to highlight that *Bacillus subtilis* is a spore-forming bacterium capable of withstanding harsh environmental conditions, including the extremely alkaline pore solution of fresh concrete (pH 12–13). Similar survival mechanisms of *Bacillus* species in cementitious environments have been reported in the literature [38]. In this study, the bacteria predominantly exist in a dormant endospore state, which confers resilience to mixing stressors and restricted nutrition supply. Instead of multiplying right after casting, the microorganisms stay inactive in the hardened matrix. Localized environmental changes that happen when cracks occur, and moisture gets in, allow spores to germinate and microbial-induced calcium carbonate precipitation (MICP) to happen, as shown before [43]. This delayed activation mechanism ensures long-term self-healing functionality without compromising fresh concrete performance.

### 3.2. Fresh and Hardened State Densities of Concrete Mix

**Figure 4:**
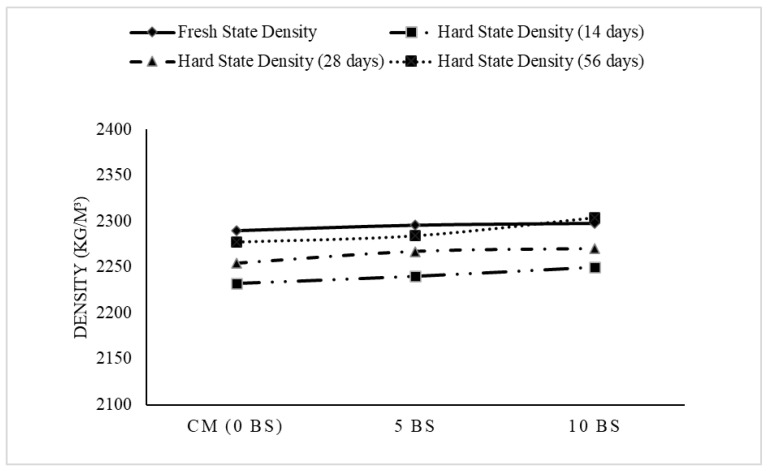
Variation in fresh and hardened concrete density with different percentages of bio-based admixture (BBA) for different curing durations.

Figure 4 shows the results of the fresh and dry-state density tests for concrete containing 0%, 5%, and 10% *Bacillus subtilis* solution, demonstrating a slight increase in density with the addition of *Bacillus subtilis* solution. The fresh state density conducted as per ASTM C138 [44]. The gradual increase suggests that the incorporation of *Bacillus subtilis* solution has a minimal yet noticeable effect on the weight of the fresh concrete, possibly due to the additional solid content or interaction of the admixture with the concrete matrix [45]. Similarly, the dry state density, measured according to [46], followed a similar trend. The slight increase in dry density indicates that the *Bacillus subtilis* admixture contributes to improved compactness and reduced porosity of the hardened concrete [25]. This behavior could be attributed to the admixture filling micro voids or enhancing the hydration process, thereby leading to denser concrete [25]. Overall, the results suggest that the addition of *Bacillus subtilis* solution as an admixture to concrete does not adversely affect the density of concrete but rather promotes a slight increase in both fresh and hardened states, which is favorable for the material’s strength and durability.

### 3.3. Air Content

**Figure 5:**
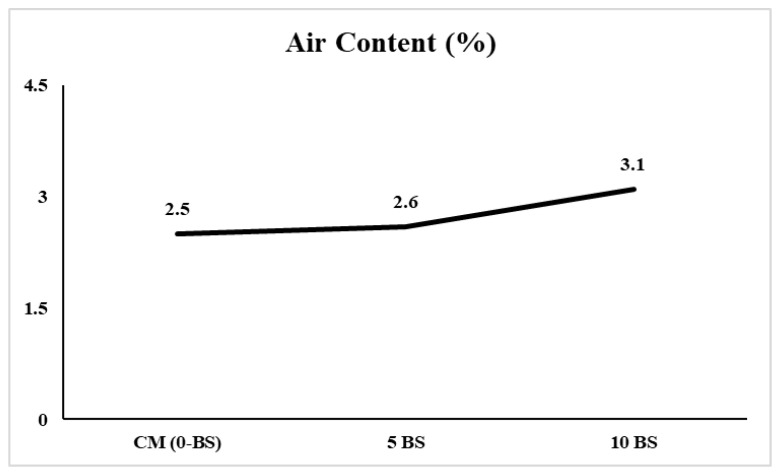
Variation in measured air content of fresh concrete mixes incorporating 0%, 5%, and 10% *Bacillus subtilis*.

Air content of fresh concrete was measured in accordance with ASTM C173 [47]. The control mix (CM, 0-BS) exhibited an air content of 2.5%, which increased marginally to 2.6% for the 5-BS mix and further to 3.1% for the 10-BS mix, as shown in Figure 5. All measured values fall within the typical range for non-air-entrained normal-weight concrete, indicating that the incorporation of the bacterial solution did not induce excessive or unstable air entrainment. The slight increase in air content with increasing bacterial dosage can be attributed to the presence of organic constituents and metabolites within the bacterial solution, which may promote limited air stabilization during mixing. Similar modest increases in air content have been reported in previous studies employing the direct addition of bacterial cultures or growth media to concrete mixes. Importantly, the observed increase remained below the threshold commonly associated with intentionally air-entrained concrete (typically 4–7%), suggesting that the bacterial admixture did not adversely affect fresh concrete compactness. Despite the gradual rise in air content, no detrimental effects on fresh or hardened concrete performance were observed. This indicates that the entrapped air was likely present in the form of small, discontinuous voids that did not compromise matrix integrity. The air content results, therefore, confirm that *Bacillus subtilis* incorporation through direct water replacement produces only minor changes in fresh-state air characteristics while maintaining acceptable concrete quality.

### 3.4. Workability of Fresh Concrete

In order to determine the impact of bacterial inclusion on the behavior of fresh concrete, the workability of the concrete mixtures was assessed using the slump test in compliance with ASTM C143 [48]. The recorded slump values for the control mix (CM 0 BS) and the bacterial concrete mixtures (5 BS and 10 BS) are shown in Table 5. The control mix demonstrated a slump of 78 mm, typical of concrete with a water-to-cement ratio of 0.50, which falls under the medium-to-high workability range typically reported for conventional concrete mixtures [49]. The addition of *Bacillus subtilis* solution led to a minor decrease in slump, with measurements of 72 mm and 67 mm recorded for the 5 BS and 10 BS mixes, respectively. The slight reduction in workability is due to the presence of bacterial cells and organic nutrients in the bacterial suspension, which may moderately elevate the viscosity of the mixing water and affect the dispersion of cement particles. Comparable behavior has been shown in previous studies [50]. However, the reduction in slump remained within acceptable limits for normal concrete placement and compaction. These results indicate that replacing a portion of the mixing water with bacterial solution does not significantly compromise fresh concrete workability, and all mixes maintained adequate consistency for practical construction applications.

## 4. Visual Inspection

**Figure 6:**
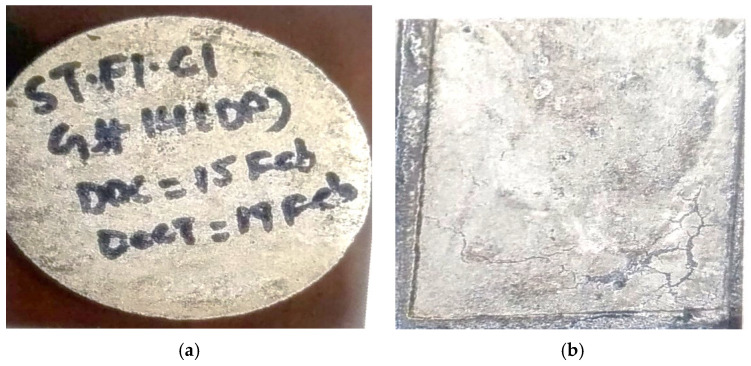
Calcite formation (**a**) Specimen-CM 0 BS (28 days), (**b**) Specimen-5 BS (24 h).

The visual inspection of the bacterial concrete specimens after 24 h of casting revealed the formation of a distinct white calcite layer on the surfaces of those incorporating *Bacillus subtilis* solution, which was absent in the control mix (CM 0 BS). This layer was notably more prominent in the 5 BS specimen (Figure 6b) compared to the CM 0 BS mix, where no such efflorescence was observed on the CM surface (Figure 6a). The 5 BS specimen is presented here as a representative bacterial-modified sample for qualitative comparison with the control mix. The increased dosage of BS solution appears to correlate with enhanced calcite deposition, suggesting prolonged bacterial viability within the calcium-rich concrete matrix, which facilitates microbial-induced calcite precipitation (MICP) and serves as a direct indicator of successful biomineralization. Even though a separate surface image of the 10 BS specimens at the 24 h stage was not obtained, the dose-dependent enhancement linked to increased bacterial content is supported by the mechanical, durability, and XRD data discussed in the next sections. As discussed in the literature, the present observation is consistent with the findings of [45]. In their investigation, visual examination performed 24–48 h after casting revealed the formation of a distinct white impermeable calcite layer exclusively on the surfaces of concrete specimens containing *Bacillus subtilis*.

Scanning electron microscopy (SEM) analysis by [45] confirmed that calcite crystals effectively sealed pores and microcracks. Higher bacterial concentrations (up to 10^7^ CFU/mL) produced significantly thicker and more prominent calcite layers (approximately 15–20% increase in thickness compared to lower dosages), which was attributed to greater bacterial viability and enhanced urease activity within the calcium-rich cementitious matrix [45].

## 5. Mechanical Properties

### 5.1. Compressive Strength Test

**Figure 7:**
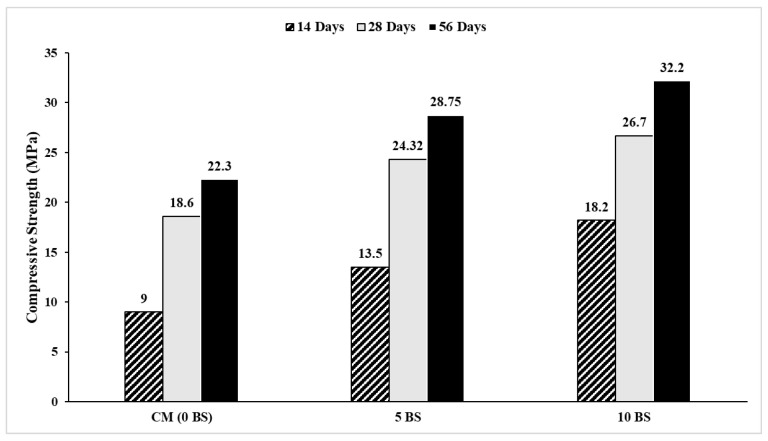
Compressive strength (14-, 28-, and 56-day curing).

Compressive strength was evaluated in accordance with ASTM C39 [51], and the results are presented in Figure 7. Concrete incorporating *Bacillus subtilis* exhibited a clear enhancement in compressive strength with increasing bacterial dosage and curing age. The control mix achieved compressive strengths of 9.0 MPa, 18.6 MPa, and 22.3 MPa at 14, 28, and 56 days, respectively, whereas the 5% bacterial mix reached 13.5 MPa, 24.3 MPa, and 28.8 MPa at the corresponding ages. The highest performance was observed for the 10% *Bacillus subtilis* mix, which achieved 18.2 MPa, 26.7 MPa, and 32.2 MPa at 14, 28, and 56 days, respectively. The strength enhancement is primarily attributed to microbial-induced calcium carbonate precipitation [18,52], which promotes pore filling and microcrack sealing, thereby increasing matrix compactness. However, the observed improvement cannot be ascribed to MICP alone.

Additional contributing mechanisms include pore refinement, improved particle packing, densification of the interfacial transition zone [53], and enhanced hydration product nucleation, all of which contribute to reduced microstructural defects and improved load transfer. The continuous increase in strength with curing age further indicates sustained cement hydration and progressive microstructural densification. Overall, the results confirm that the direct incorporation of *Bacillus subtilis,* particularly at a dosage of 10%, significantly enhances the compressive strength of concrete while maintaining structural integrity. The gradual increase in strength can be attributed to the natural hydration of cement, where calcium silicate hydrates (C-S-H) continue to form and densify the concrete matrix. Recent studies [54] support these findings, highlighting that higher bacterial concentrations, such as 10%, maximize strength gain due to increased microbial activity and void filling. However, it is important to note that excessively high bacterial content (above 10–12%) may compromise workability and lead to inconsistent results. The curing duration also plays a critical role in strength development [55]. For all formulations, the compressive strength increased significantly from 14 days to 56 days, indicating that the extended curing period allows for continued hydration of cement and prolonged microbial activity. The sustained presence of *Bacillus subtilis* within the concrete facilitates long-term calcium carbonate formation, further enhancing the densification of the concrete matrix and reducing microstructural defects [27]. In conclusion, the addition of *Bacillus subtilis* significantly improves the compressive strength of concrete, with the 10% bacterial content yielding the highest performance. These findings align with current literature and demonstrate the potential of microbial concrete as a sustainable and durable material for construction applications.

Importantly, the observed strength enhancement should not be interpreted solely as a consequence of pore filling. The continuous increase in compressive strength with bacterial dosage is accompanied by a pronounced modification of post-peak response, indicating that microbial activity alters the internal damage progression rather than merely increasing matrix density. This distinction is critical, as strength gains arising from densification alone typically correlate with increased brittleness, whereas the bacterial concrete exhibited enhanced strain capacity and gradual stress decay, suggesting a fundamentally different microstructural response to loading.

### 5.2. Stress–Strain Behavior

**Figure 8:**
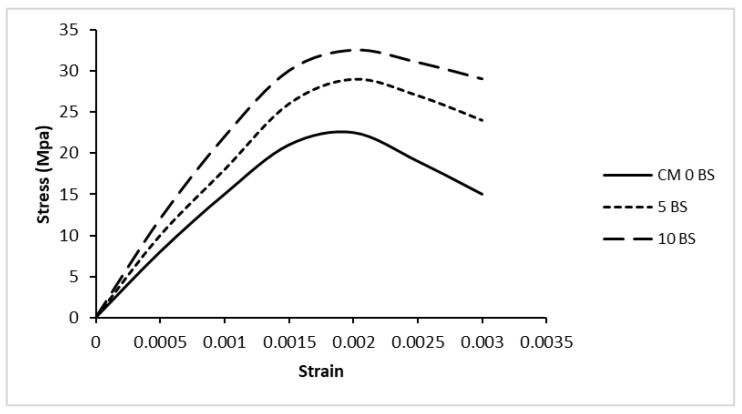
Stress strain response (compression).

**Figure 9:**
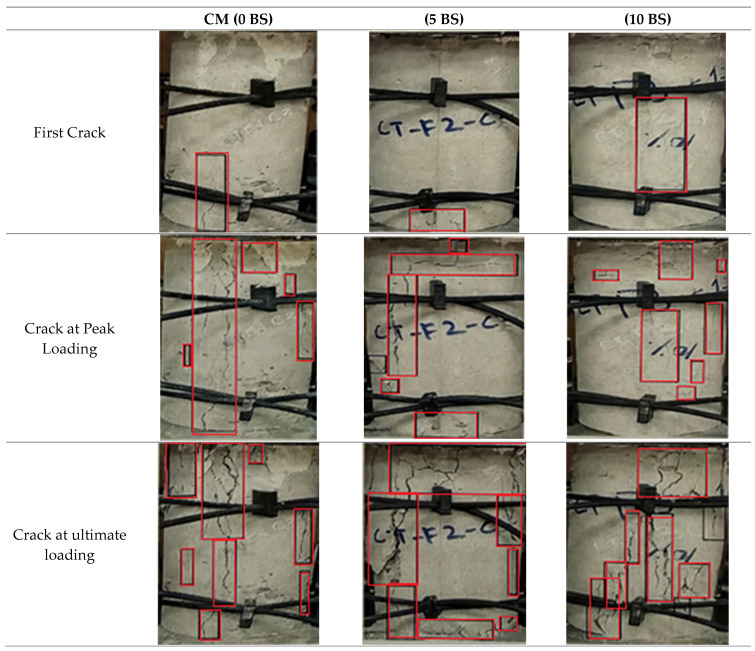
Crack propagation in cylindrical specimen.

Figure 8 illustrates the compressive stress–strain response of the control and bacterial concrete mixtures, while Figure 9 presents the crack propagation patterns observed in the cylindrical specimens during compression testing. The curves demonstrate how concrete usually behaves when it is compressed. There is an initial linear elastic area, then a nonlinear ascending branch that goes up to the peak stress, and finally a descending branch that shows how cracks spread as the material softens. The control mix (CM 0 BS) reached a peak stress of about 22.3 MPa at a strain of about 0.0019–0.0020. The initial portion of the curve is nearly linear, representing the elastic response of concrete before significant microcrack formation. After reaching the peak stress, the control specimen showed a relatively steeper descending branch, indicating brittle failure behavior typical of conventional concrete.

Conversely, the incorporation of *Bacillus subtilis* resulted in improved stress–strain behaviors. The 5 BS mixture obtained a peak stress of about 28.75 MPa, whereas the 10 BS mixture reached a peak stress of about 32.2 MPa. The bacterial concrete mixtures also had somewhat greater peak strains and a more gradual post-peak descending branch than the control specimen. This shows that they can deform better and delay the development of cracks.

The enhancement in stress–strain behavior can be attributed to microbial-induced calcium carbonate precipitation (MICP), which promotes calcite deposition within pores and microcracks, leading to pore refinement and densification of the interfacial transition zone. This enhanced microstructure facilitates stress transfer between the cement matrix and aggregates, leading to increased load-carrying capacity and greater ductility. Comparable trends have been documented in previous studies. Rao et al. (2017) [56] reported that bacterial concrete exhibits higher stress values at the same strain levels compared with conventional concrete, along with increased elastic modulus and improved mechanical performance due to calcite precipitation within the concrete matrix.

The elastic modulus of each mixture was calculated using the empirical relationship

(1) $$E_{c}=4700\sqrt{fc^{\prime }}$$

where $E_{c}$ is the elastic modulus (MPa), and $fc^{\prime }$ = compressive strength (MPa).

The calculated stress–strain parameters are summarized in Table 6.

The crack propagation patterns shown in Figure 8 support the stress–strain behavior observed in Figure 7. The control specimen exhibited early crack initiation and localized vertical cracking, whereas the bacterial concrete specimens showed delayed crack formation and more distributed cracking patterns. At peak and ultimate loading stages, bacterial mixes developed multiple finer cracks compared with the dominant crack in the control specimen, indicating improved stress redistribution and crack resistance. The larger area under the stress–strain curves of bacterial mixes also suggests enhanced compressive toughness and energy absorption capacity due to microbial-induced calcium carbonate precipitation (MICP) within the concrete matrix.

### 5.3. Split Tensile Strength Test

**Figure 10:**
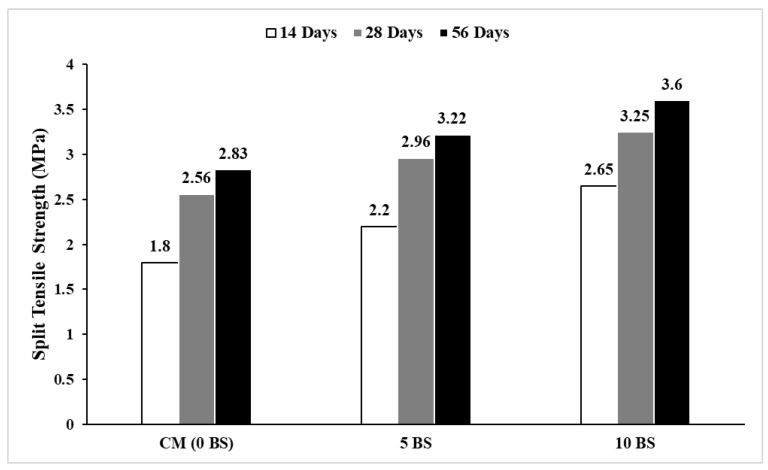
Split tensile strength (14-, 28-, and 56-day curing).

Split tensile strength was carried out in accordance with ASTM C496 [57]. The split tensile strength results, as shown in Figure 10 for concrete specimens, demonstrate a clear improvement in tensile performance with increasing bacterial content and extended curing durations. The trends observed align with the compressive strength findings and highlight the role of microbial activity in enhancing the tensile properties of concrete [20]. The precipitation of CaCO_3_ effectively acts as a filler material, healing microcracks and reducing voids, which in turn leads to improved tensile performance [18,20,52]. The significant increase in tensile strength highlights the effectiveness of higher bacterial concentrations in strengthening the concrete matrix. This improvement can be attributed to prolonged hydration of cement and sustained microbial activity within the matrix [55,58]. The continued precipitation of calcium carbonate during extended curing periods further heals cracks and densifies the matrix, improving the tensile properties over time. These results further validate the potential of microbial concrete as a durable and sustainable material for construction applications.

### 5.4. Flexural Strength, Energies Absorbed, and Toughness Index Under Flexural Loads

**Figure 11:**
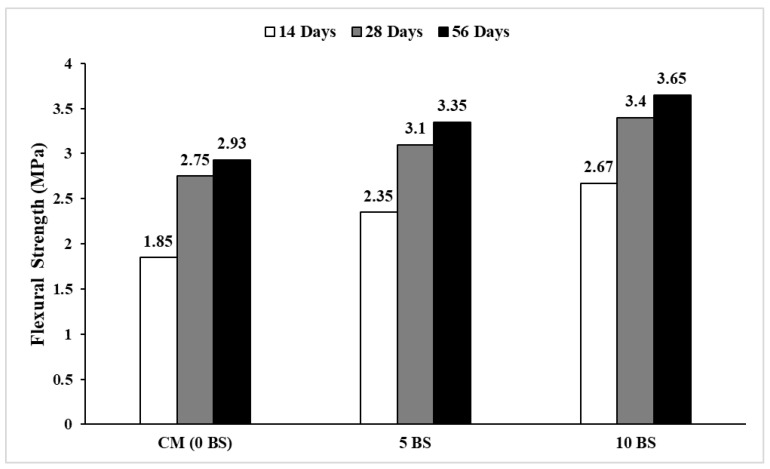
Flexural strength (14-, 28-, and 56-day curing).

**Figure 12:**
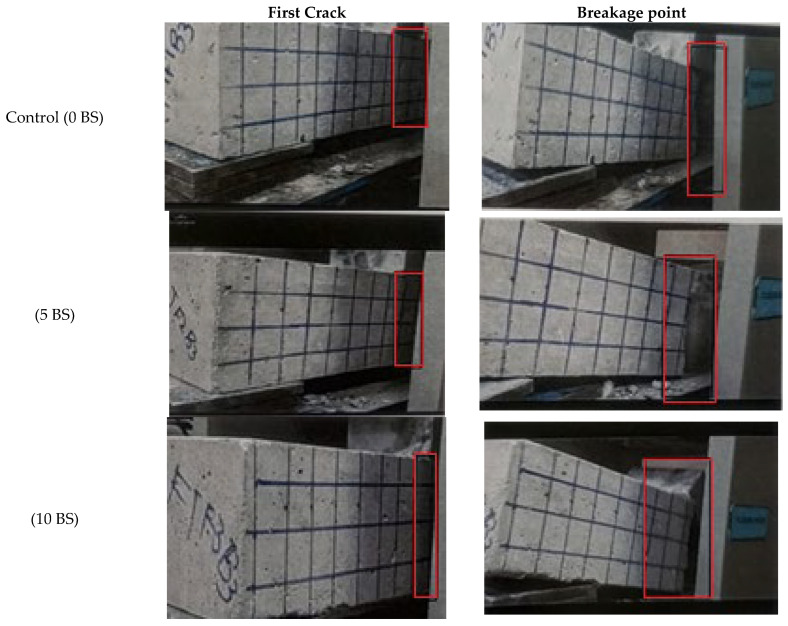
Crack propagation in beam specimens.

The flexural performance parameters of concrete mixes are summarized in Figure 2 and Figure 11 and Table 7, and the flexural strength test was conducted in accordance with ASTM C78 [59], while the corresponding crack propagation patterns under flexural loading are shown in Figure 12. The control mix exhibited a flexural strength of 2.93 MPa, accompanied by relatively low post-cracking energy absorption and a flexural toughness index (FTI) of 0.56, indicating brittle behavior typical of conventional concrete [49]. The incorporation of *Bacillus subtilis* resulted in a clear enhancement in flexural performance. Flexural strength increased to 3.35 MPa and 3.65 MPa for the 5% and 10% bacterial mixes, respectively. More notably, the post-crack absorbed energy (FPCE) increased from 2.054 kN·mm in the control mix to 2.76 kN·mm and 3.35 kN·mm, while the total absorbed energy (FTAE) increased to 3.88 kN·mm for the 10% *Bacillus subtilis* mix. Consequently, the flexural toughness index increased significantly from 0.56 to 0.84, reflecting improved post-cracking load-carrying capacity and deformation tolerance. The crack propagation patterns shown in Figure 12 corroborate these results. The control specimen exhibited early crack localization and rapid crack widening, whereas bacterial-modified specimens showed delayed crack initiation and the development of multiple finer cracks with reduced crack widths. This distributed cracking behavior, particularly evident in the 10% *Bacillus subtilis* mix, indicates enhanced crack-bridging and stress redistribution capacity, which directly contributes to the observed increase in energy absorption and toughness [24,29].

The enhancement in flexural toughness has significant implications beyond structural performance. Increased post-cracking energy absorption indicates improved crack-bridging capacity, which directly influences transport properties by limiting the formation of continuous crack networks. This observation provides a mechanistic explanation for the concurrent reduction in chloride ion penetration and sorptivity observed in bacterial concretes, highlighting the role of damage tolerance rather than strength alone in governing durability.

## 6. Durability

### 6.1. Rapid Chloride Ion Penetration Test (RCPT)

**Figure 13:**
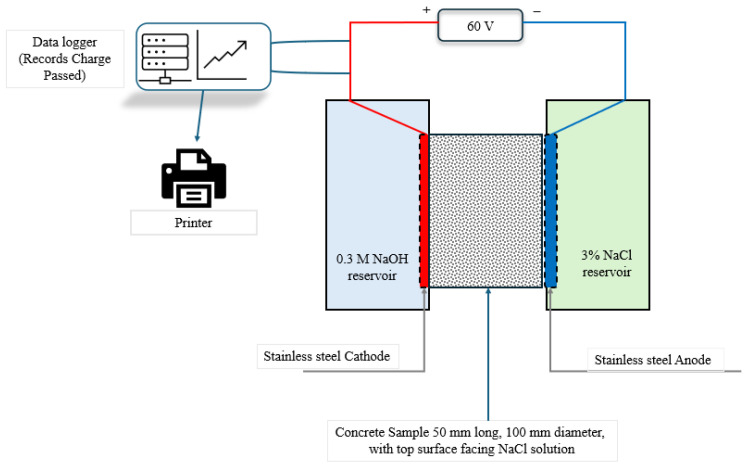
Test setup for chloride ion penetration ASTM C1202 [60].

The Rapid Chloride Ion Penetration Test (RCPT), according to ASTM C1202 [60], assessed concrete’s chloride ion resistance. Cylindrical specimens (100 mm × 200 mm) were moist-cured in saturated limewater at 23 ± 2 °C for 28 days. Disk specimens (50 ± 2 mm thick) were removed, epoxy-sealed, vacuum-saturated, and evaluated at 60 ± 0.1 V for 6 h. One side of each specimen was exposed to 3.0% NaCl and the other to 0.3 N NaOH. The test setup is shown in Figure 13 and the results are summarized in Table 8. The inclusion of *Bacillus subtilis* significantly reduced the charge transferred in a dose-dependent fashion. The 5% bacterial mixture recorded 2700 Coulombs (29.9% reduction; moderate permeability); however, the 10% mixture exhibited 1650 Coulombs (57.1% reduction) relative to the control mixture, which is classed as low permeability according to [60]. The decrease in chloride ions correlates with a denser pore shape that obstructs connecting capillary channels, therefore restricting chloride transport [26,61,62]. The findings indicate that bacterial inclusion, especially at a 10% dose, markedly improves resistance to chloride intrusion, hence enhancing the durability of concrete in chloride-exposed conditions.

The decrease in chloride ion penetrability cannot be solely ascribed to diminished porosity, as samples displaying increased ductility and dispersed cracking had the lowest charge transmission. This indicates that bacterial inclusion inhibits crack-assisted chloride transport by restricting crack connectivity and breadth during prolonged loading. Thus, the enhancement of durability in bacterial concrete is determined by the synergistic effects of pore refinement and altered damage processes, underscoring the significance of post-cracking behavior in evaluating long-term performance.

### 6.2. Tests on Transport Properties (Capillary Sorptivity)

**Figure 14:**
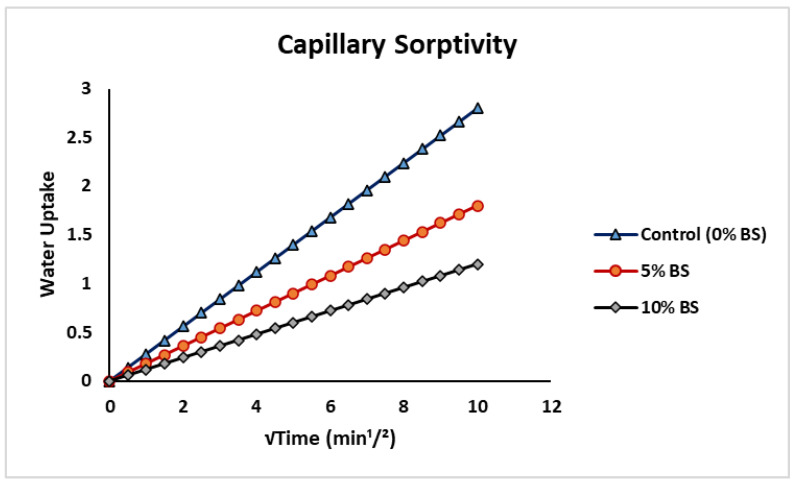
Capillary water absorption (sorptivity) curves for concrete mixes with 0%, 5%, and 10% *Bacillus subtilis* solution (cured for 28 days).

Capillary water absorption was assessed using ASTM C1585-20 [63]. After conditioning in a bacterial solution or lime-saturated water for 6 days, cylindrical specimens were air-dried at 20 °C and 40% relative humidity for 10 days. Test specimens were submerged in shallow water with only the bottom surface exposed and the lateral surfaces sealed to allow unidirectional water penetration. Mass gain was recorded at intervals, and cumulative water absorption (*I*, mm) was calculated:

(2) $$I=\frac{M_{t}}{A\times D}$$

where *Mₜ* is the mass change at time *t* (g), *A* is the exposed surface area (mm^2^), and *D* is the density of water (g/mm^3^). Sorptivity (*S*) was obtained from the slope of the linear portion of the plot of *I* versus √*t*. The capillary sorptivity results are presented in Figure 14. The 0% *Bacillus subtilis* mixture had the highest water absorption, attaining roughly 2.8 mm at √*t* = 10–11 min^1/2^, which correlates to a sorptivity coefficient of about 0.28 mm/min^1/2^. The introduction of *Bacillus subtilis* led to a significant, dose-dependent decrease in water absorption. The 5 BS mix demonstrated an absorption of around 1.8 mm (sorptivity = 0.18 mm/min^1/2^), but the 10 BS mix displayed the lowest uptake at approximately 1.2 mm, indicating a 50–60% decrease in sorptivity compared to the control. *Bacillus subtilis* promotes calcite deposition in capillary pores and microcracks, thereby diminishing pore connectivity and enhancing the density of the cement matrix and interfacial transition zone [10,24]. Similar reductions in sorptivity (30–70%) have been reported in previous studies on *Bacillus*-based bio-concrete systems [25,27]. The significant reduction in capillary water absorption enhances the superior mechanical performance and diminished chloride ion penetrability identified in this investigation. The results demonstrate improved resistance to moisture penetration and harsh substances, underscoring the efficacy of *Bacillus subtilis* as a bio-based additive for creating durable, low-permeability concrete.

## 7. X-Ray Diffraction (XRD) Analysis

**Figure 15:**
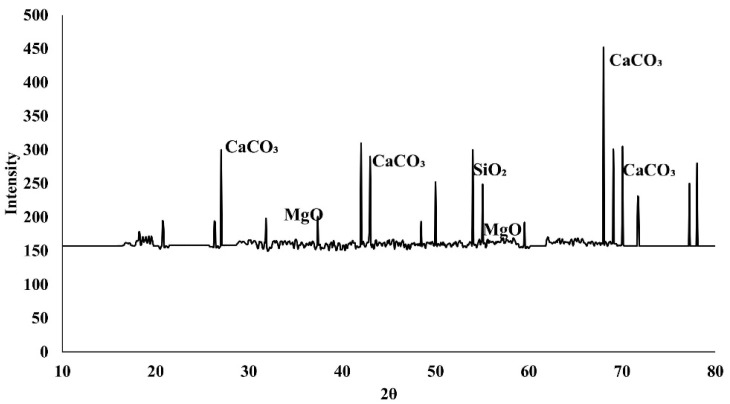
X-ray diffraction pattern of concrete containing 10% *Bacillus subtilis* after 56 days of curing, showing characteristic calcite (CaCO_3_) peaks associated with microbial-induced calcium carbonate precipitation.

X-ray diffraction (XRD) analysis was performed on powdered concrete samples containing 10% *Bacillus subtilis* after 56 days of curing to find crystalline phases linked to microbial activity. The investigation utilized a high-resolution diffractometer with Cu Kα radiation (λ = 1.5406 Å), conducted across a scanning range of 10–80° (2θ) with a step size of 0.02° and a scan speed of 2°/min. The XRD pattern depicted in Figure 15 exhibits unique diffraction peaks associated with calcite (CaCO_3_), thus affirming the presence of microbial-induced calcium carbonate precipitation (MICP) inside the concrete matrix. Significant calcite peaks were seen at roughly 2θ = 29.4°, 39.4°, 43.1°, and 47.5°, which are indicative reflections of crystalline calcite. Additionally, peaks associated with SiO_2_ and MgO were identified, stemming from the cementitious and aggregate phases. The existence of distinct calcite peaks in the bacterial concrete offers direct microstructural evidence of MICP, corroborating the noted enhancements in mechanical performance and transport qualities. The deposition of CaCO_3_ in pores and microcracks enhances matrix densification, refines pores, and decreases permeability, as seen by the sorptivity and RCPT data.

## 8. Conclusions

- The direct incorporation of *Bacillus subtilis* (1.9 × 10^7^ cells/cm^3^) by water substitution yielded significant enhancements in mechanical and durability characteristics without negatively impacting fresh qualities.
- The characteristics of fresh concrete were only slightly affected by the presence of bacteria. The air content rose somewhat from 2.5% (control) to 3.1% (10% BS), remaining below permissible limits for non-air-entrained concrete. The fresh and hardened densities showed a marginal increase, signifying enhanced matrix compactness while maintaining workability.
- Compressive strength significantly increased with bacterial dosage and cure duration. At 28 days, the compressive strength increased from 18.6 MPa (control) to 26.7 MPa (10% BS), indicating a rise of roughly 43.5%. At 56 days, strength rose from 22.3 MPa to 32.2 MPa, reflecting a 44.4% improvement. The 10% blend exhibited the greatest peak stress and enhanced post-peak performance.
- The flexural strength rose from 2.93 MPa (control) to 3.65 MPa (10% BS) at 56 days, indicating an enhancement of roughly 24.6%. The post-crack absorbed energy rose from 2.054 kN·mm to 3.35 kN·mm, representing an approximate 63% increase, while the total absorbed energy escalated from 2.054 kN·mm to 3.88 kN·mm, indicating an approximate 89% increase. The flexural toughness index rose from 0.56 to 0.84, indicating a 50% enhancement in post-cracking load-bearing capability and deformation resilience.
- The Rapid Chloride Penetration Test (ASTM C1202) indicated a decrease in charge passed from 3850 Coulombs (moderate–high permeability) for the control sample to 1650 Coulombs (low permeability) for the 10% mix, reflecting a 57.1% drop in chloride ion penetrability.
- Capillary sorptivity diminished from 0.28 mm/min^1/2^ (control) to 0.18 mm/min^1/2^ (5% BS) and subsequently to roughly 0.12 mm/min^1/2^ (10% BS), indicating a 50–60% drop in water absorption.
- X-ray diffraction (XRD) analysis of the 10% bacterial mixture revealed distinct calcite (CaCO_3_) peaks at 2θ ≈ 29.4°, 39.4°, 43.1°, and 47.5°, offering direct microstructural evidence of microbial-induced calcium carbonate precipitation (MICP).
- Collectively, enhancements in compressive strength (approximately 44%), flexural strength (approximately 25%), toughness index (50%), chloride resistance (57%), and sorptivity reduction (up to 60%) illustrate that MICP functions synergistically with pore refinement and densification of the interfacial transition zone, yielding improved strength, ductility, and durability performance.
- The 10% *Bacillus subtilis* dosage achieved an optimal equilibrium between mechanical enhancement and durability improvement, validating the practicality and adaptability of direct water-replacement bacterial incorporation for producing low-permeability, crack-resistant, and chloride-resistant concrete.
- To gain a deeper knowledge of microstructural development, additional long-term studies are recommended. These should involve thorough studies using the Rapid Chloride Penetration Test (RCPT) to assess the distribution of chloride ions and mapping utilizing Energy Dispersive Spectroscopy (EDS) to examine elemental composition and how it impacts the development of crack width.

## Figures and Tables

**Table 1:** Chemical and physical characterization of OPC.

Parameters	OPC
SiO_2_	19.19
TiO_2_	0.29
Al_2_O_3_	4.97
Fe_2_O_3_	3.27
MnO	0.04
MgO	2.23
CaO	65
Na_2_O	0.58
K_2_O	0.51
P_2_O_5_	0.08
LOI (Loss on Ignition)	3.84
Particle size (D50), µm	16.4
BET surface area, in^2^/lb	0.57 × 10^6^
Density, lb/in^3^	0.114

**Table 2:** Physical properties of fine and coarse aggregate.

Property	ASTM Standards	Fine Aggregate	Coarse Aggregate
Finesse Modulus	ASTM 136-6 [33]	2.79	6.49
Nominal Size	ASTM 136-6 [33]	-	10
Specific gravity (SSD)	ASTM 127-1 [34]	2.685	2.665
Specific Gravity	ASTM 127-1 [34]	2.647	2.642
Apparent Specfic Gravity	ASTM 127-1 [34]	2.751	2.704

**Table 3:** Composition of growth media.

Tryptone	NaCl	Yeast	Distilled Water	LB Broth
2.5 g	2.5 g	1.75 g	25 g	1 g

**Table 4:** Concrete mix proportions and bacterial solution replacement strategy.

Mix ID	Cement (kg/m^3^)	Fine Aggregate (kg/m^3^)	Coarse Aggregate (kg/m^3^)	Mixing Water (kg/m^3^)	*Bacillus subtilis* Solution (kg/m^3^)	Water Replacement (%)
CM (0-BS)	380	765	1526	160	0	0
5-BS	380	765	1526	152	8	5
10-BS	380	765	1526	144	16	10

Note: The bacterial solution replaced an equivalent mass of mixing water to maintain a constant water-to-cement ratio (w/c = 0.50) for all formulations. Calcium lactate was dissolved in the liquid phase prior to mixing to ensure uniform nutrient availability for microbial-induced calcium carbonate precipitation.

**Table 5:** Slump values of concrete mixtures containing different percentages of *Bacillus subtilis* solution.

Mix ID	Bacterial Solution Content (%)	Water-to-Cement Ratio	Slump (mm)
CM (0 BS)	0	0.50	78
5 BS	5	0.50	72
10 BS	10	0.50	67

**Table 6:** Stress–strain parameters of concrete mixtures.

Mix	Peak Stress (MPa)	Peak Strain	Elastic Modulus (GPa)
CM (0 BS)	22.3	0.0020	22.2
5 BS	28.75	0.0021	25.2
10 BS	32.2	0.0022	26.6

**Table 7:** Flexural strength, deflection at first crack, energy absorption parameters, and flexural toughness index of concrete mixes containing 0%, 5%, and 10% *Bacillus subtilis*.

Parameters	CM (0 BS)	5 BS	10 BS
Flexural Strength (FS, MPa)	2.93	3.35	3.65
Deflection at first crack (Δ_0_, mm)	0.067	0.029	0.111
Pre-crack absorbed energy (FAPE, kN·mm)	0.054	0.029	0.533
Post-crack absorbed energy (FPCE, kN·mm)	2.054	2.76	3.35
Total absorbed energy (FTAE, kN·mm)	2.054	2.76	3.88
Flexural toughness index (FTI)	0.56	0.61	0.84

Note: FS = Flexural strength; Δ_0_ = deflection at first crack; FAPE = pre-crack absorbed energy; FPCE = post-crack absorbed energy; FTAE = total absorbed energy; FTI = flexural toughness index.

**Table 8:** Chloride ion penetrability based on charge passed.

ID	Charge Passed (Coulombs)	Permeability
CM	3850	Moderate to High
5 BS	2700	Moderate
10 BS	1650	Low

## Data Availability

The original contributions presented in this study are included in the article. Further inquiries can be directed to the corresponding author.
